# A Study Evaluating the Functional Outcomes of Mason Type III and IV Radial Head Fractures Treated With a Radial Head Prosthesis

**DOI:** 10.7759/cureus.66898

**Published:** 2024-08-14

**Authors:** Ashwin Deshmukh, Rahul Agrawal, Swaroop Solunke, Abhishek Nair, Ankit Barosani

**Affiliations:** 1 Orthopaedics, Dr. D. Y. Patil Medical College, Hospital and Research Centre, Dr. D. Y. Patil Vidyapeeth (Deemed to be University), Pune, IND

**Keywords:** mayo elbow performance index, elbow dislocation, modified mason’s classification, radial head arthroplasty, radial head fracture

## Abstract

Objective

This research aimed to assess the functional result of type III and IV radial head fractures that were treated using a radial head prosthesis.

Methods

A retrospective investigation was conducted on 70 patients with type III and IV radial head and neck fractures, as classified by Mason. The patients were hospitalized and received treatment at the Orthopaedics Department, where they had radial head prosthesis surgery for three years.

Results

Among the total of 70 cases, 42 (60%) cases were below the age of 40, while 28 (47%) cases were over 40 years. The average age was 36.4 years. The maximum age recorded was 54 years, while the lowest age recorded was 30 years. The female population outnumbered the male population. The majority of instances (42, 60%) were attributed to falls, while the remaining cases were caused by road traffic accidents (RTAs). Out of the total 70 instances, 52 cases (74.28%) exhibited right-side dominance, whereas 18 cases (25.72%) exhibited left-side dominance. Within our case study group, 56 (80%) cases fell under modified Mason's classification type Ill, totaling 56 instances. The remaining 20% of the cases, amounting to 11 cases, were classified as modified Mason's classification type IV. Among the 70 patients, 55 cases (78.58%) did not have any ligamentous damage, whereas seven (10%) cases had lateral ulnar collateral ligament (LUCL) injury and eight (11%) cases had medial collateral ligament (MCL) injury. The P value for flexion, extension, pronation, and supination was shown to be very significant. Out of the total, 47 (67%) instances had an MEPI score (Mayo Elbow Performance Index) of more than 90, indicating exceptional performance. In addition, 16 cases (22.85%) had an MEPI score ranging from 75 to 89, which is considered a good result. Lastly, seven cases (10%) had an MEPI score ranging from 60 to 74, indicating a fair result.

Conclusion

The use of a radial head prosthesis is considered a viable option for managing severe and irreparable fractures of the radial head. Effective outcomes hinge on meticulous preoperative planning, skilled intraoperative techniques, and intensive postoperative rehabilitation. These elements collectively contribute to achieving consistent and favorable results in patients undergoing this surgical intervention.

## Introduction

Fractures affecting the radial head and neck constitute approximately 1.7-5.4% of all fractures and represent about 33% of all elbow fractures [1-4]. Typically, these fractures occur due to falls where individuals land on their outstretched arms [5,6]. The radial head plays a crucial role in the movement of the elbow joint, contributing significantly to flexion, extension, pronation, and supination movements [7]. Structurally, it provides essential support against the lateral stress on the elbow and helps maintain stability throughout the length of the forearm [8]. Typically, the management of radial head fractures depends on the specific classification of the fracture. The modified Mason classification is widely accepted as the standard categorization system for articular fractures of the radial head. Mason type I fractures involve marginally (<2 mm) or non-displaced radial head fractures. Type II fractures are defined by marginal sector fractures that exhibit some degree of displacement(>2 mm). Type III fractures encompass complete and comminuted radial head fractures. Type IV fractures involve radial head fractures associated with elbow dislocation [9]. Radial head fractures constitute approximately 3% of all fractures and are the most common type of elbow fracture in adults [10,11]. The choice of treatment approach, whether nonoperative or surgical, is determined by factors such as the extent of the fracture, the degree of displacement, patient-specific factors, and functional goals. Radial head fractures commonly result from falls where the individual lands on an outstretched arm with the forearm pronated. The severity of these fractures can range from simple to complex, impacting the stability of the elbow joint [12]. Generally, fractures isolated to the radial head without associated fractures or ligament injuries tend to be stable, even if displaced by more than 2 mm [11].

The stability of the fracture and the treatment approach depend on factors such as the degree of displacement, involvement of adjacent structures, and the patient's functional needs. Operative treatment for radial head fractures, whether through open reduction and internal fixation (ORIF) or radial head prosthetic replacement, aims to prevent elbow joint subluxation or dislocation by restoring stability and proper alignment of the radio-humeral joint [12]. In the cases of elderly patients with complex isolated fractures, radial head resection is often considered. This approach may also be suitable for fracture-dislocations without a coronoid fracture and no signs of longitudinal or medial instability, especially in patients with low physical demands [13-15]. This tailored approach ensures optimal management based on the specific characteristics of the fracture and the patient's functional needs. Radial head prostheses restrict the upward displacement of the radius bone under vertical pressure applied to the forearm. They prevent lateral bending of the elbow and posterior instability by maintaining robust contact within the radio-capitellar joint, mimicking the function of the native radial head. This helps facilitate the proper healing and function of the medial collateral ligament, interosseous ligament, and distal radio-ulnar joint. Given these considerations, many orthopedic surgeons opt for radial head arthroplasty as the primary treatment for Mason type III and IV radial head fractures [16]. This approach aims to restore elbow joint stability and functionality effectively, especially in cases where conservative management or other surgical options may not provide optimal outcomes.

## Materials and methods

A retrospective investigation was conducted on 40 patients with type III and IV radial head and neck fractures, as classified by Mason. The patients were hospitalized at the Department of Orthopaedics of Dr. D. Y. Patil Medical College, Hospital & Research Centre, Pune, India, and received treatment including the use of a radial head prosthesis for three years. 

The study included patients who had severely shattered fractures of the radial head and neck, specifically those classified as type III and IV. Patients who had reached skeletal maturity for the surgical procedure were included. Informed consent was taken from each patient. The exclusion criteria included patients with open fractures, additional fractures around the elbow, cases involving infection, and pediatric patients with fractures of the radial head and neck.

**Figure 1:**
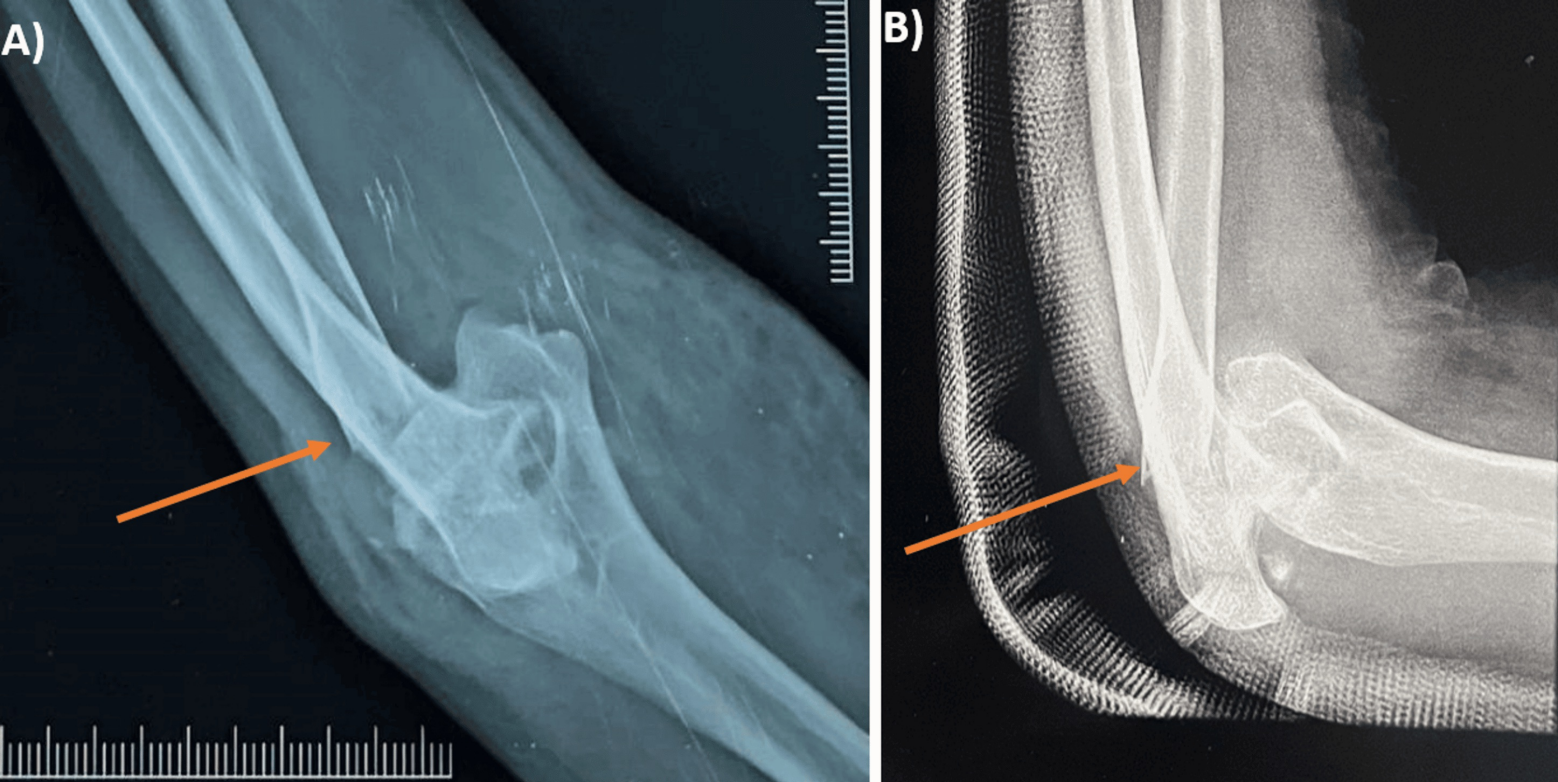
Preoperative X-ray A) Preoperative radiograph showing the anteroposterior (AP) view of the left elbow with Mason type IV radial head fracture. B) Preoperative radiograph showing the lateral view of the left elbow with Mason type IV radial head fracture.

**Figure 2:**
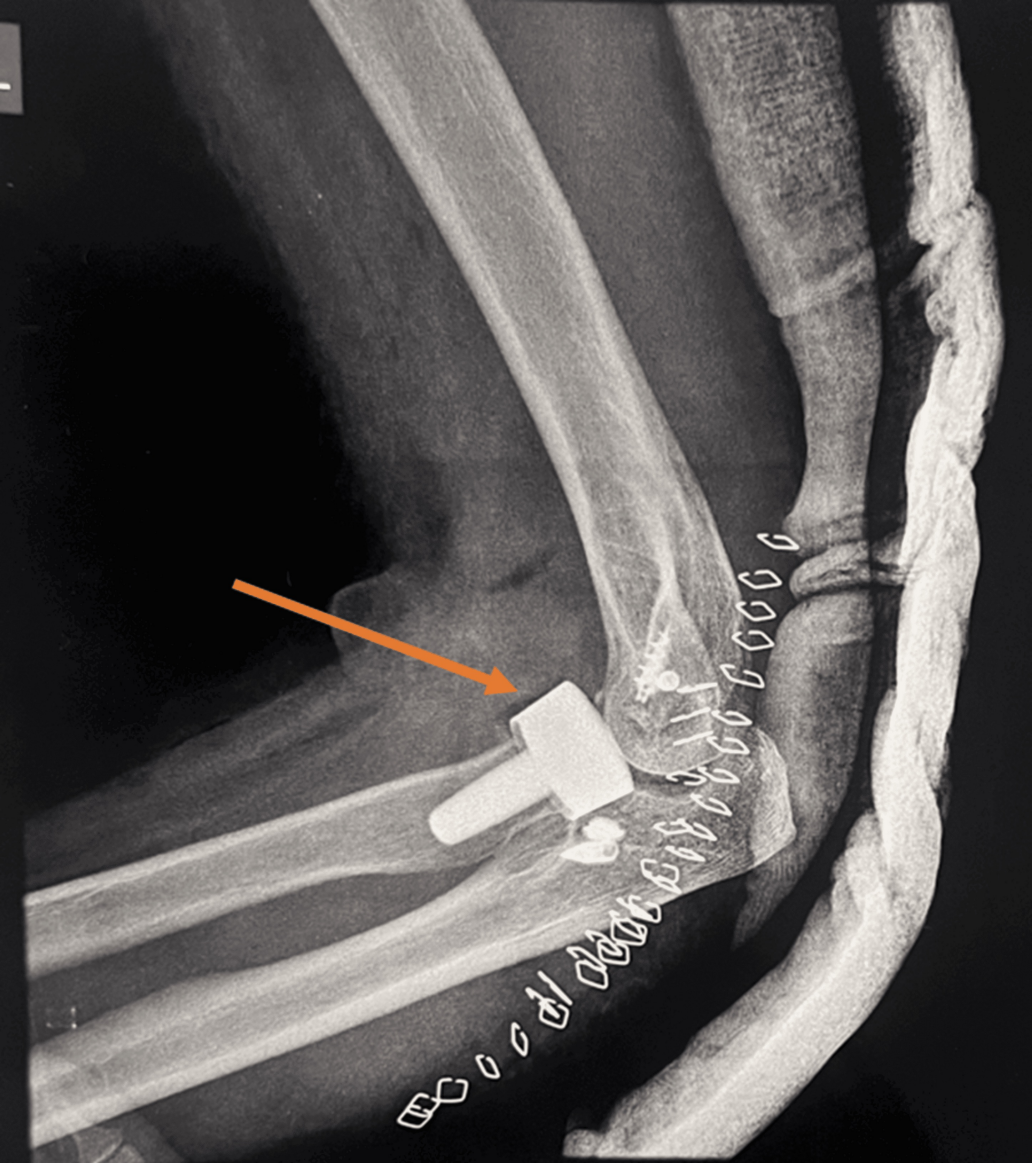
Postoperative radiograph showing Mason type IV radial head fracture treated with a radial head prosthesis

Before surgery, patients had an evaluation upon admission. This evaluation included obtaining a full history, which included gathering information on the cause of damage and the patient's symptoms. In addition, a comprehensive clinical examination was conducted. Anteroposterior and lateral radiographs of the afflicted and the normal limbs were captured on the opposite side. Immobilization was administered for initial therapy in the form of an above-elbow slab. All necessary standard investigations were completed and the patient's fitness for preoperative anesthesia was assessed. The patient had a surgical procedure to replace the radial head with a radial head prosthetic (Figures 1, 2). Monopolar prostheses were utilized and were press-fit implanted using Kaplan’s lateral access and Kocher’s anconeus approach to the humeroradial joint. The prostheses were procured from Orthotech India Private Limited.

Following surgery, the patients underwent a standardized postoperative care protocol at our institution. They received an antibiotics course and anti-inflammatory medications for three days as per our institutional guidelines to prevent infection and manage inflammation. Active flexion and extension exercises for the elbow, encompassing the full range of motion (ROM), were initiated three days post-surgery. The patients wore a collar and cuff to support and protect the elbow during the exercises. At night, they used a static progressive extension splint to maintain an extension. Patient evaluations were conducted systematically, measuring the ROM at two and six weeks post-operation. In addition, the Mayo Elbow Performance Score (MEPS) was utilized to assess stability and overall functioning during the final follow-up appointment.

The statistical data analysis was performed using IBM SPSS Statistics, Version 24.0 (released 2016, IBM Corp., Armonk, NY). A significance level of p < 0.05 was chosen to determine the statistical significance of the study findings.

## Results

Among the total of 70 cases, 42 (60%) cases were below the age of 40, while 28 (40%) cases were over 40. The average age was 36.4 years. The maximum age was 54 years, while the lowest age recorded was 30 years. The female population outnumbered the male population. In the majority of instances, almost 42 (60%) were attributed to falls, while the remaining cases were caused by road traffic accidents (RTAs). Out of the total number of cases, 52 instances (74.28%) exhibited right-side dominance, whereas 18 cases (25.72%) exhibited left-side dominance (Table 1).

**Table 1:** Demographic details

Age in years	N	%
<40	42	60
>40	28	40
Gender		
Male	24	34.28
Female	46	65.72
Mode of injury		
RTA	21	30
Fall	42	60
Dominant side		
Right	52	74.28
Left	18	25.72

Out of the 70 cases in our study group, 56 (80%) were classified as modified Mason's type III fractures and 14 (20%) as type IV fractures. Among the 70 cases, 55 (78.58%) had no ligamentous injuries. However, seven (10%) cases involved injury to the lateral ulnar collateral ligament (LUCL), and eight (11%) cases had medial collateral ligament (MCL) injuries (Table 2).

**Table 2:** Mason's classification and associated injury

Mason’s classification	N	%	
Type III	56		80
Type IV	14		20
Associated injury			
LUCL	7		10
MCL	8		11.42
None	55		78.58

At two weeks post-operation, the mean flexion was 76.34 degrees, which increased to 118.32 degrees by six weeks. During this period, the mean extension deficit improved from 25 degrees to 10 degrees. In addition, the mean pronation at two weeks was 20 degrees, rising to 65.15 degrees by the sixth week. Supination also showed notable improvement, with an average of 35 degrees at two weeks post-op, increasing to 70.36 degrees by week six. These changes in flexion, extension, pronation, and supination were statistically significant, as indicated by their p-values (Table 3).

**Table 3:** Post-operative flexion, extension, pronation, and supination at the second and sixth post-op weeks

Parameter	2nd week	6th week	P-value
Flexion			
ROM (Degrees)	76.34±22.68	118.32±15.95	<0.0001
Extension			
ROM (Degrees)	25±10.72	10±11.55	<0.005
Pronation			
ROM (Degrees)	20±4.56	65.15±7.43	<0.0001
Supination			
ROM (Degrees)	35±7.23	70.36±7.30	<0.0001

Forty-seven patients (67.15%) had MEPI scores (Mayo Elbow Performance Index) >90, indicating exceptional results; 16 cases (22.85%) had 75-89, indicating good results; and seven cases (10%) had 60-74, indicating fair results (Table 4).

**Table 4:** Mayo Elbow Performance Index (MEPI)-wise distribution

MEPI	N	%
<60 (Poor)	0	0
60-74 (Fair)	7	10
75-89 (Good)	16	22.85
>90 (Excellent)	47	67.15

## Discussion

The most important findings of this study aimed at evaluating the use of a radial head prosthesis as a viable option for managing severe and irreparable fractures of the radial head. Modified Mason type I and II fracture treatment typically involves conservative management or ORIF. Modified Mason type I and II fractures can often be managed nonoperatively or with ORIF. However, the optimal surgical approach for modified Mason type III and IV fractures remains debated in the literature, largely due to the challenges posed by persistent instability resulting from these more complex injuries [17].

This was a multicentric study with a standard approach, technique, type of implant, and post-op protocol and rehabilitation. In our study of 70 cases, 42 (60%) patients were under 40 years old, and 28 (40%) were 40 years or older, with an average age of 36.4 years. The youngest patient was 30 years old, while the oldest was 54 years old. These findings are consistent with those of Chien et al. [18], who analyzed 13 patients with radial head fractures and reported a similar average age of 36.4 years. In our study, the female population outnumbered the male population., but in the research by Kulkarni et al. [19], out of 30 patients, 60% were male and 40% were female.

The majority of radial head fractures (42, 60%) were attributed to falls, while the remaining cases resulted from RTAs. According to Kadam et al. [20], the majority of individuals sustained fractures from falls onto an extended hand, followed by injuries from RTAs, and least from assault. In our study, 74.28% of fractures showed dominance on the right side, with 25.72% on the left. This aligns closely with findings from Kulkarni et al. [19], who reported 53.33% of fractures on the right and 46.67% on the left. Surgical management of comminuted and irreparable radial head fractures poses significant challenges due to associated ligament injuries and resulting elbow instability. While radial head excision has historically been favored, recent advancements in radial head prostheses have greatly enhanced surgical outcomes.

The radial head serves crucial roles as a stabilizer against valgus forces and in transmitting axial force loads during elbow flexion [21]. It also restricts varus and external rotatory movements [22]. Comminuted Mason type III and IV fractures often coincide with other elbow injuries, such as capitellum and coronoid fractures, as well as medial and lateral ligament and interosseus membrane disruptions [23,24]. In our cohort of 70 cases, 80% were classified as modified Mason type III and the remaining 20% as Type IV. Among these patients, 78.58% had no ligamentous damage, while 10% had an injury to the lateral ulnar collateral ligament (LUCL) and 11.42% to the medial collateral ligament (MCL). Postoperatively, the mean flexion angle at two weeks was 76.34 degrees, which improved to 118.32 degrees by six weeks. The average extension deficit reduced from 25 degrees at two weeks to 10 degrees at six weeks post-surgery. Pronation averaged 20 degrees at two weeks and increased to 65.15 degrees at six weeks, while supination averaged 35 degrees initially and reached 70.36 degrees by six weeks post-surgery. Statistical analysis showed highly significant p-values for flexion, extension, pronation, and supination outcomes.

In our study of 47 cases, 67.15% achieved an MEPI score greater than 90, indicating excellent outcomes. In addition, 22.85% scored between 75 and 89, classified as good outcomes, while 10% scored between 60 and 74, indicating fair performance. Comparatively, Kulkarni et al. [19] reported on 30 cases where 66.67% showed great results (MEPI > 90), 26.66% had good results (MEPI 75-89), 3.33% had fair results (MEPI 60-74), and another 3.33% had poor outcomes. On the other hand, Kadam et al. [20] observed outcomes in 18 patients, with 72% achieving outstanding results, 17% showing good outcomes, and 11% experiencing fair results. Furthermore, out of 10 cases, 80% had no complications, while 10% experienced infection and another 10% dealt with elbow stiffness.

These findings suggest favorable outcomes overall, emphasizing the effectiveness of radial head prosthesis in treating type III and IV radial head fractures, albeit with varying degrees of success reported across different studies.

## Conclusions

Radial head prostheses show favorable functional results in cases with modified Mason's type III and IV radial head fractures, with reduced complications and the ability to begin movement earlier in the recovery process. Effective care of type III and IV radial head injuries relies on preoperative surgical planning. Further evaluation of the efficacy of radial head prosthesis in radial head and neck fractures necessitates a long-term follow-up with a larger sample size.
